# Physiological and transcriptional approaches reveal connection between nitrogen and manganese cycles in *Shewanella algae* C6G3

**DOI:** 10.1038/srep44725

**Published:** 2017-03-20

**Authors:** Axel Aigle, Patricia Bonin, Chantal Iobbi-Nivol, Vincent Méjean, Valérie Michotey

**Affiliations:** 1 Aix-Marseille Université, CNRS, Université de Toulon, IRD, MIO UMR 110, 13288, Marseille, France; 2 Aix-Marseille Université, CNRS, BIP Bioénergétique et Ingénierie des Protéines UMR 7281, 13402, Marseille, France

## Abstract

To explain anaerobic nitrite/nitrate production at the expense of ammonium mediated by manganese oxide (Mn(IV)) in sediment, nitrate and manganese respirations were investigated in a strain (*Shewanella algae* C6G3) presenting these features. In contrast to *S. oneidensis* MR-1, a biotic transitory nitrite accumulation at the expense of ammonium was observed in *S. algae* during anaerobic growth with Mn(IV) under condition of limiting electron acceptor, concomitantly, with a higher electron donor stoichiometry than expected. This low and reproducible transitory accumulation is the result of production and consumption since the strain is able to dissimilative reduce nitrate into ammonium. Nitrite production in Mn(IV) condition is strengthened by comparative expression of the nitrate/nitrite reductase genes (*napA, nrfA, nrfA-2*), and rates of the nitrate/nitrite reductase activities under Mn(IV), nitrate or fumarate conditions. Compared with *S. oneidensis* MR-1, *S. algae* contains additional genes that encode nitrate and nitrite reductases (*napA*-α and *nrfA-2*) and an Outer Membrane Cytochrome (OMC)(*mtrH*). Different patterns of expression of the OMC genes (*omcA, mtrF, mtrH* and *mtrC*) were observed depending on the electron acceptor and growth phase. Only gene *mtrF-2* (SO1659 homolog) was specifically expressed under the Mn(IV) condition. Nitrate and Mn(IV) respirations seem connected at the physiological and transcriptional levels.

In a large part of the sediment floor, organisms use terminal electron-accepting molecules, alternatives to oxygen for energy generation^1^. The most abundant molecules comprise NO_3_^−^, Mn(IV), Fe(III), SO_4_^2−^ or organic matter resulting from fermentation. In sedimentary systems, their repartition is often stratified on a permanent or seasonal basis. Just below the oxic zone, the suboxic zone is characterized by high concentrations of oxidized inorganic compounds, such as nitrate, manganese oxides (Mn(IV)) and iron oxy/hydroxides (Fe(III)) that are in close proximity^1^. Nitrate is generally known to be generated in aerobiosis by nitrification from ammonium, which results from the mineralization of organic matter. Iron and manganese are commonly encountered in the Earth’s crust. Water percolating through soil and rock can dissolve minerals containing iron and manganese and hold them in solution. After oxidation, mainly by water-soluble oxygen, the metal oxides precipitate and settle into the sediment.

Biogenic redox reactions have been recognized as paramount for global biogeochemical cycles. From the geochemical point of view, manganese-oxides are commonly described as powerful oxidants capable of modifying or creating new pathways of oxidation in the N cycle^2^,^3^,^4^,^5^,^6^,^7^,^8^,^9^. Luther *et al.,* (1997) have shown strong links between Mn and N cycles and have proposed several putative reactions causing short-cuts in the N cycle^6^. Biogeochemical evidence has been found for such reactions, for example, the enhanced N_2_ production in the presence of Fe(III) or Mn(IV) in marine sediment samples^10^. Furthermore, laboratory incubation experiments with Atlantic coastal sediment showed a significant increase in N_2_O production upon the addition of Mn(IV)^11^. The same trend was observed in mangrove sediment from India: N_2_O production was higher in sediment subjected to ferromanganese mining operations in comparison to a pristine site^12^. Among NH_4_^+^ oxidation processes, the role of Mn(IV) compounds in alternative anaerobic metabolism has often been suggested to explain the presence of unexpected bursts of nitrite/nitrate in deep layers of sediment^2^.

However, due to the complex recycling of redox species, these processes are difficult to discern and therefore are poorly understood. Javanaud *et al.,* (2011) isolated a denitrifying strain belonging to the genus *Marinobacter* from Atlantic coastal sediments and used it to demonstrate N_2_ production from NH_4_^+^ 13. Anaerobic nitrification–denitrification in the presence of manganese oxides was suggested, and a putative formation of nitrate or nitrite at the expense of ammonium by this manganese oxide reducing strain was proposed^13^.

*Shewanella oneidensis* MR-1 is the most-described and well-known strain capable of dissimilatory metabolism of manganese oxides^14^. The genus *Shewanella* is known for its versatile electron-accepting capacity, which allows the coupling of the decomposition of organic matter to the reduction of the various terminal electron acceptors that these organisms encounter in their stratified environments. The model species *Shewanella oneidensis* MR-1 presents the notable properties of using more than 10 terminal electron acceptors and of transferring electrons to solid metal oxides. Currently, bacteria of the genus *Shewanella* comprise more than 50 species that inhabit a wide range of marine environments and ecosystems. In the frame of the study of anaerobic ammonium oxidation mediated by Mn(IV), a pool of *Shewanella* strains able to perform nitrate and Mn(IV) reductions was isolated from the Atlantic coastal sediment^13^. The genome of one of these strains, *Shewanella algae* C6G3, has been recently sequenced and differences in the gene organization compared with *Shewanella oneidensis* MR-1 were identified (Fig. 1A,B).

To complement information obtained from the literature on gene-encoding proteins involved in Mn(IV) respiration based on mutagenesis in *S. oneidensis* MR-1^15^,^16^,^17^, our study explores the kinetics of the expression level by qRT-PCR of the gene-encoding potential “end effectors” for Mn(IV) (*omcA, mtrC, mtrF, mtrH*) and nitrate/nitrite (*napA, nrfA*) respirations, two oxidized inorganic compounds in close vicinity in sedimentary systems during the growth of *S. algae* C6G3. Two other genes, SA002_00782 and SA002_01180, which encode a putative decaheme *c*-type cytochrome MtrF and a putative cytochrome *c*-552 precursor, respectively, were also monitored. During growth with Mn(IV) or nitrate as the electron acceptor, the kinetics of nitrate, nitrite, Mn(II) production or consumption were investigated to obtain system-level insights on anaerobic NO_2/3_^−^ production in the presence of Mn(IV). Moreover, the gene expression levels were compared under the two respiration conditions, growth with fumarate being used as control. Our results demonstrate a transitory nitrite accumulation during growth with Mn(IV) in agreement with the concomitant expression of nitrate/nitrite reductase genes and nitrate and nitrite reductase activities. Concerning the OMC, their expression is growth-phase dependent and peaked at the end of the exponential phase under the nitrate and Mn(IV) conditions in contrast to that observed for the control with fumarate. Except for *mtrH*, all OMC tested genes presented higher expressions in nitrate or Mn(IV) compared with the control (fumarate). However, only the new gene *mtrF-2* was specifically expressed during the entire growth period in Mn(IV) compared with nitrate and fumarate conditions.

## Results and Discussion

### Nitrate and Mn(IV) respiratory gene analysis, classical and “new genes” identification

**Figure 1:**
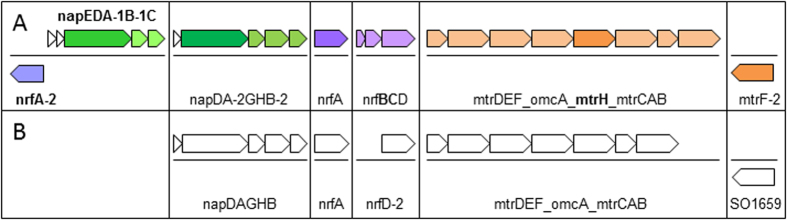
Comparative organization of genes involved in Nitrate and Mn(IV) respiratory systems in *S. algae* C6G3 and *S. oneidensis* MR-1. (**A**) Presentation of the potential operons carrying genes of NAP systems (dissimilatory nitrate reduction, DNR), NRF systems (dissimilatory nitrite reduction into ammonium, DNRA) and Mtr systems (reduction of external electron acceptors). The presented genes correspond to the organization of *S. algae* C6G3 genome check manually (BLASTP against *S. algae* strain ACDC and JCM 21037 on JGI IMG/ER, *nrfA-2* = SA002_01180, *mtrH* = SA002_00064 and *mtrF-2* = SA002_00782 and *mtrEFC*), or automatically annotated (the other genes). (**B**) Presentation of corresponding operons carrying the genes of interest in *S. oneidensis* MR-1. Note that *nrfA-2, napEDA-1B-1C, nrfBC* and *mtrH* are absent in the *S. oneidensis* MR-1 genome (bold).

From the sequence of the genome of *S. algae* C6G3 (GenBank ID JPMA00000000 (JPMA01000001-JPMA01000043)^18^) the genes encoding NAP, NRF and Mtr systems giving electrons to nitrate, nitrite or ion metal oxide, respectively and ensuring the electron transfer from the cytoplasm to the periplasm and extracellular compounds was proposed for this strain (Fig. 1A). Except for *S. oneidensis* MR-1 and *S. denitrificans* OS217, the genome of the majority of the species belonging to the genus *Shewanella* including *S. algae* C6G3 and *S. algae* ACDC present two isoforms of the NAP operon, Nap-α and Nap-β^19^,^20^ insuring the reduction of nitrate into nitrite by a periplasmic nitrate reductase (NapA) (Fig. 1A,B).

The nitrite reduction step into ammonium was performed by a periplasmic cytochrome *c* nitrite reductase NrfA (Fig. 1A,B)^21^. In addition to the NAP-α operon, a gene potentially involved in the transformation of the nitrogenous element was identified in the genome of *Shewanella algae* C6G3 (formally SA002_01180) and annotated as formate-dependent nitrite reductase, periplasmic cytochrome *c*‐552 subunit with KO:K03385 (EC 1.7.2.2) and COG3303 according to the JGI IMG/ER platform. After sequencing and verification of the sequence integrity, this gene was herein referenced as *nrfA-2*. The BLASTp of the sequence on NCBI revealed the presence of *nrfA-2* homologs among the sequenced genomes only in the genus *Shewanella* and *Ferrimonas* with an occurrence of 61% (n = 29) and 100% (n = 4), respectively (see Fig. S1 in the supplemental material). As for the Nap-α operon, *nrfA-2* is absent in the *S. oneidensis* MR-1 genome (Fig. 1B). The role of the cytochrome *c*-552 is unknown. The study of Clark *et al.,* (2013)^22^ on *S. algae* ACDC excluded its involvement in nitrite reductase activity because the presence of the *nrfA-2* homolog in the *S. algae* ACDC genome did not permit the nitrite reduction activity, when the *nrfA* gene is inactivated by the presence of a transposon^22^. Other authors have also noted the absence of any role of this gene in the respiration of the nitrate or nitrite in *S. piezotolerans* WP3^20^.

The respiratory external electron acceptor machinery (mainly Fe(III) and Mn(IV)) has been extensively studied in the model strain *S. oneidensis* MR-1. These studies have identified the effectors of the electron transport chain from the cytoplasm to the cell surface where the external electron acceptor is reduced via the Mtr system^23^. The Mtr system consists of a group of transmembrane and periplasmic cytochrome encoded genes organized in an operon^24^ comprising from the cytoplasm to the outer membrane: (I) the periplasmic tetraheme cytochrome *c* CymA^20^,^25^,^26^,^27^, (II) the cytochromes MtrAB and their homologues MtrDE and (III) the extracellular deca-haem *c*-type cytochromes of the OmcA/MtrC family^28^ (OMC) responsible for the reduction of extracellular metal oxides and flavins^29^,^30^,^31^,^32^.

In *S. algae* C6G3, the Mtr operon is composed of 8 genes *mtrDEF-omcA-mtrH-mtrCAB* (Fig. 1A). The *mtrH* gene is absent in *S. oneidensis* MR-1 Mtr operon (Fig. 1B). This extracellular deca-haem *c*-type outer-membrane -cytochrome^24^,^33^,^34^ is found in only 23% of the *Shewanella* strains tested (n = 29, data not shown), and it is referenced in *S. halifaxensis* HAW- EB4 (Shal_2783) and *S. piezotolerans* WP3 (swp_3277)^33^,^34^. Extensive studies on the genome of *S. oneidensis* MR-1 have also revealed the presence of 42 potential *c*-type cytochromes (soluble periplasmic proteins, cytoplasmic membrane proteins and outer membrane lipoproteins) distributed on its genome^35^. Two out of the 5 identified as outer membrane lipoproteins are external to the Mtr system: SO1659 (decaheme *c*) and SO2931 (diheme *c*). Investigations into the genome of *S. algae* C6G3 have identified only the homolog of SO1659 as SA002_00782. This gene has been annotated as a decaheme c-type cytochrome, OmcA/MtrC family in the JGI IMG/ER platform and as *mtrF* in the RAST platform; thus, it was herein referenced as *mtrF-2* to distinguish it from *mtrF* of the Mtr operon. The gene *mtrF*-2 is distributed in 100% of the tested strains belonging to the *Shewanella* genus (n = 29; see Fig. S2 in the supplemental material); this ubiquity suggests a paramount role.

### Connection between NO_3_^−^ and Mn(IV) respiration on the physiological level through nitrite production associated with Mn(IV) respiration

**Figure 2:**
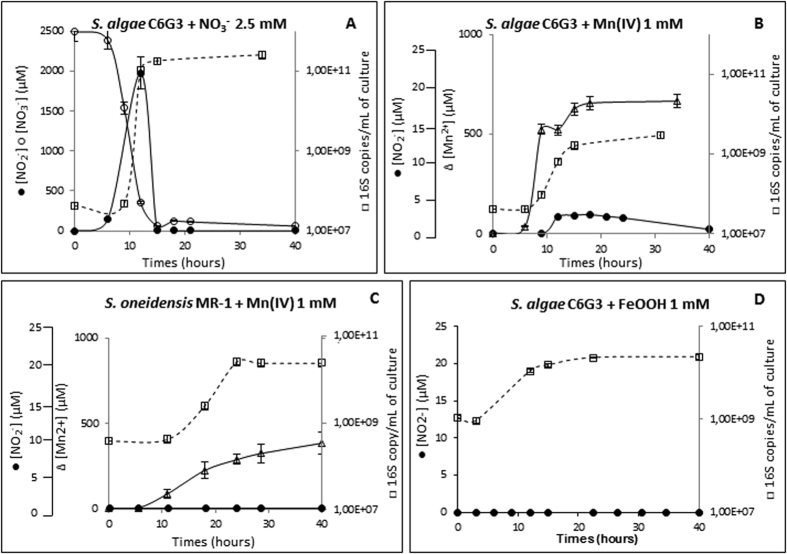
(**A–D**) Kinetic of growth of *S. algae* C6G3 and *S. oneidensis* MR-1 in anaerobiosis and in the presence of nitrate or Mn(IV) or FeOOH as electron acceptor in minimal medium with lactate as electron donor. Bacterial growth (□) and dissolved nitrite (●), nitrate (○) and manganese (Δ) concentrations.

*Shewanella algae* C6G3 is capable of using nitrate or Mn(IV) as the sole electron acceptor under anaerobic conditions^18^, with a generation time 2-fold shorter (0, 64 h vs. 1, 32 h), and a culture yield 25-fold higher for the nitrate condition (Fig. 2A,B).

For the growth of *S. algae* C6G3 with nitrate, there was a rapid consumption of nitrate correlated with nitrite accumulation which was rapidly consumed (Fig. 2A), the same trend was observed for *S. algae* ATCC 51192 (see Fig. S3A in the supplemental material). For *S. algae* C6G3 growth on Mn(IV), Mn^2+^ production is observed to reach approximately 70% of the initial concentration of Mn(IV). In contrast to culture of *S. oneidensis* MR-1 (Fig. 2C), there is a low but reproducible nitrite accumulation (reaching ~2 μM) during the exponential phase of growth for the *S. algae* strains C6G3 and ATCC 51192 (Fig. 2B; see Fig. S3B in the supplemental material). During the stationary phase of growth, the nitrite concentration is maintained then decreases from 18 h to 40 h with a concomitant 3-fold increase in the number of 16 S rDNA (1.63 × 10^9^ genes/mL at 18 h; 6.2 × 10^9^ genes/mL at 40 h) (Fig. 2B). Neither nitrate nor nitrous oxide was detected in the cultures of *S. algae* in the presence of Mn(IV). The nitrite burst was not observed in media without cells (data not shown) or in cultures with Fe(III) as the electron acceptor (Fig. 2D for 1 mM Fe(III), data not shown for 3 mM Fe(III)).

**Figure 3:**
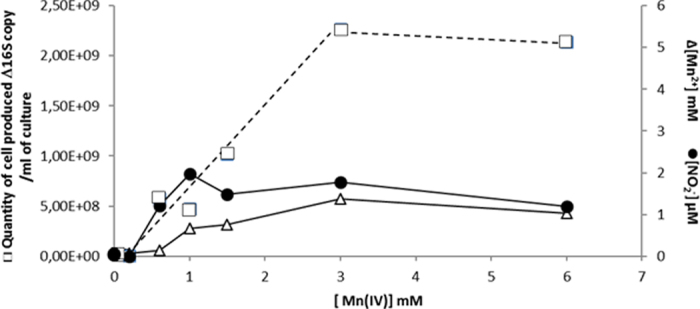
Impact of initial Mn(IV) concentration on quantity of *S algae* C6G3 cells produced (Final quantity minus initial quantity □) and Mn^2+^ concentrations at the end of the culture (Δ) and on maximal dissolved nitrite accumulation during growth (●).

The quantity of *S algae* C6G3 cells generated during growth under manganese oxide reducing conditions and in minimal medium with lactate as the sole carbon and energy source, was proportional to initial Mn(IV) concentration in the range 1–3 mM (Fig. 3) whereas a plateau value was observed for 6 mM of Mn(IV) probably due to an inhibitory effect. Reduction of Mn(IV) changed the color of the precipitates from dark brown to whitish rosy-red. For initial Mn(IV) concentration of 3 or 6 mM, soluble Mn(II) quantified at the end of the growth never exceeded 1.7 mM indicating that most Mn(II) was associated with the solid phase. The mineral formed was presumably MnCO_3_.

In the culture with 3 mM of Mn(IV), the oxidation of 5.4 mM of lactate into acetate (4.15 mM) and CO_2_ requires at least the reduction of 10 mM of Mn(IV) to Mn(II).


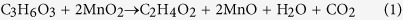


The culture experienced electron acceptor limitation, since only 3 mM Mn(IV) were available.

In consequence, under these conditions we can assume that another electron acceptor was involved in lactate oxidation. The small amount of nitrite (2 μM) accumulated in media containing NH_4_Cl as the nitrogen source during manganese reduction suggested the possibility that anaerobic ammonium oxidation into nitrite or nitrate occurred and their subsequent utilization as electron acceptors and reduction into ammonium.

Investigations of the impact of the Mn(IV) concentration on nitrite production by cultures of *S. algae* C6G3 have been also performed in a range comprised between 0.05 to 6 mM (Fig. 3). A nitrite accumulation has been observed for the initial concentrations of the metal oxide above 0.6 mM, whereas no nitrite could be detected for 0.05 and 0.2 mM. No correlation between the nitrite accumulation and the Mn(IV) initial concentration was observed (n = 15, p < 0.05, Fig. 3)). Due to its rapid production and consumption, nitrite could be a reactive short-lived intermediate and its level of accumulation depends only on the difference in the rates of production and consumption.

Several studies have highlighted nitrate and nitrite production in sediment slurries incubated under anaerobic conditions upon the addition of Mn(IV)^13^,^36^, suggesting a putative production of nitrate for at least some strains. Taking into account the above data, the nitrite production in culture of *S. algae* strains C6G3 and ATCC 51192, thus seems likely to be of biotic origin. The nitrite concentration (~2 μM) observed in a pure culture of *S. algae* strains C6G3 and ATCC 51192 is in the same range as that observed in Mn(III/IV)-rich marine sediment^2^ (~5 μM of NO_2/3_^−^) or in an enriched mesocosm^36^ (3.3 to 4.9 μM of NO_2/3_^−^). Furthermore, as observed in our study, several authors have also noted the transient nature of this accumulation in sediment^13^,^36^. However, the Mn(III/IV) concentrations necessary to obtain *in vitro* NO_2/3_^−^ peaks are higher than the values found in Mn(III/IV)-rich marine sediments where this phenomenon has been observed (e.g., ~0.2 mM of Mn(III/IV)^2^).

**Figure 4:**
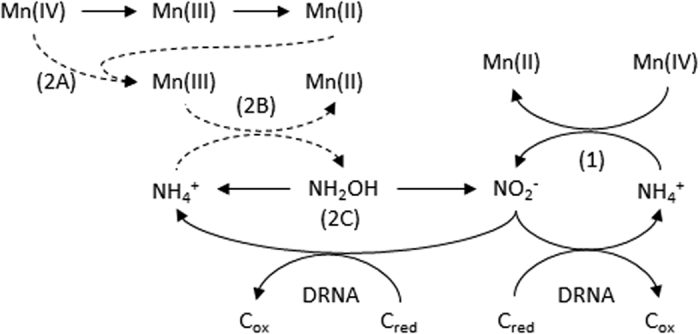
Hypotheses for the tight coupling of the nitrogen and manganese cycles in *S. algae* C6G3. The first one (1) implies a direct biotic oxidation of NH_4_^+^ by Mn(IV), whereas the second one (2 ABC) involves an abiotic generation of Mn(III) via the comproportionation reaction between Mn(IV) and Mn(II) (2A), followed by an abiotic oxidation of NH_4_^+^ by this highly reactive Mn(III) into NH_2_OH and Mn(II) (2B), and finished by a biotic disproportionation of NH_2_OH into NO_2_^−^ and NH_4_^+^ (2 C). Dotted arrow: abiotic reaction, full arrow: biotic reaction. C_ox_: oxidized electron donor, C_red_ reduced electron donor.

Two hypotheses for the tight coupling of the nitrogen and manganese cycles have been proposed (Fig. 4). The first is the direct oxidation of NH_4_^+^ to NO_2/3_^−^ via the reduction of manganese oxides (Equation 2 and pathway 1 Fig. 4) as proposed by several authors^2^,^4^,^6^,^8^ from results obtained from anoxic sediment.


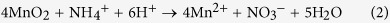


The second hypothesis involves Mn(III) which is a labile intermediate formed in the presence of Mn(IV) and Mn(II). Mn(III) is extremely unstable in solution. The available Mn(III) produced herein would catalyze NH_4_^+^ oxidation into N_2_ or NH_2_OH that would be subsequently be subjected to a biological disproportionation into NH_4_^+^ and NO_2_^−^ (pathway 2 Fig. 4), in analogy with the anoxic oxidation of H_2_S to SO_4_^2−^ previously described^37^.

Overexpression of genes encoding for enzymes associated with dissimilatory reduction of nitrate (nap) and nitrite (nrfA) and enzymatic activity of the encoding gene under manganese oxide reducing conditions could be an indirect proof of the production of nitrate/nitrite and its subsequent reduction via dissimilatory nitrate reduction into ammonium pathway.

### Expression of nitrate respiratory genes and enzymatic activity of the encoding proteins in cells grown the presence of nitrate or Mn(IV) as the electron acceptor

**Figure 5:**
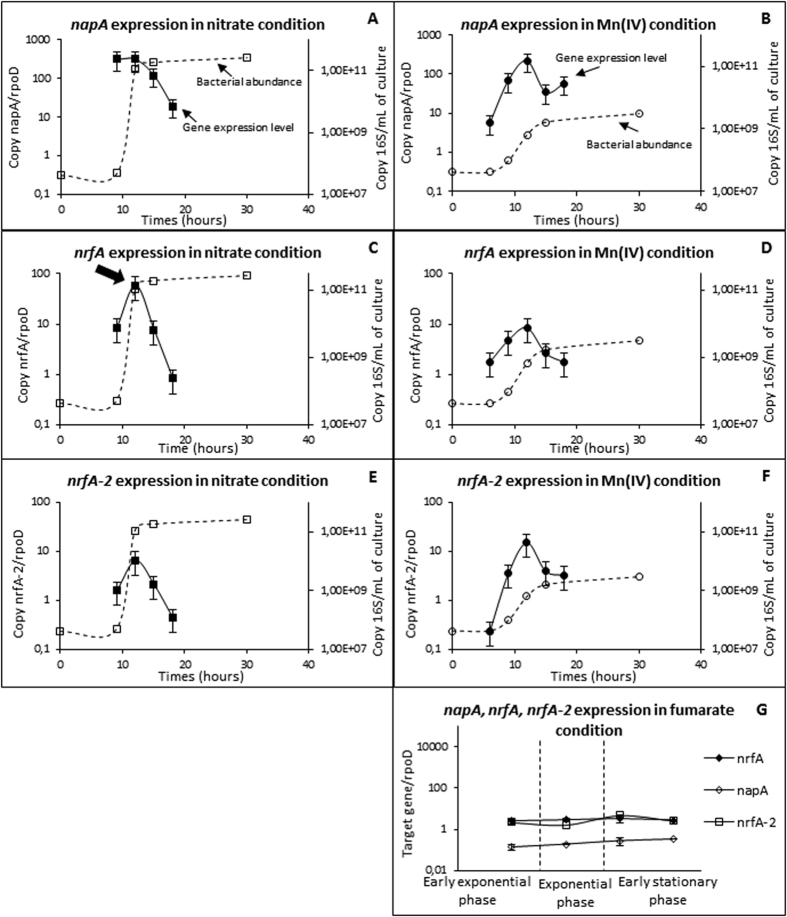
(**A–G)** *napA, nrfA and nrfA-2* gene expression level along bacterial growth with nitrate or manganese as the electron acceptor. The time of the kinetic corresponding to the nitrite peak is marked with a bold arrow. Cultures with fumarate (**G**) correspond to negative control. Standard deviation of growth curves has not been materialized for readability.

The *rpoD*-relative expression level of the genes involved in the terminal transfer of electrons to nitrate and nitrite was investigated because their gene products could be involved in the nitrite production. In *S. oneidensis* MR-1, the expression level of numerous genes has been tested by short incubation without growth in the presence of different electron acceptors^38^. In the presence of nitrate in comparison with fumarate, expression of *napA* and of *nrfA* increase of 4.9 and 20.5-fold, respectively, suggesting an induction of these genes by nitrate/nitrite, whereas in the presence of Mn(IV), *napA* and *nrfA* expression levels were close to that of the fumarate control (i.e., 1.98 and 1.24). Another study on *S. oneidensis* MR-1 has shown that *napA* is responsive to nitrate, whereas *nrfA* is responsive to both nitrate and nitrite^39^. *S. algae* C6G3 presents two isoforms of *napA* with 73% similarity on the nucleotide level. It was not possible to quantify their individual expression level with the chosen primer set^40^. In the *S. algae* C6G3 control cultures (fumarate), the *napA* and *nrfA* expression level relative to *rpoD* remained stable during growth ranging from 0.1 to 1. In the culture of *S. algae* C6G3 with nitrate, the expression level of the nitrate reductase genes *napA (napA*-α + *napA*-β) was induced during the exponential growth phase (~300 relative to *rpoD*), corresponding to the consumption of nitrate and the production of nitrite (Fig. 5A). The expression level of *napA* decreased rapidly during the stationary phase of growth as nitrate is consumed. The expression level of *nrfA* encoding the dissimilatory nitrite reductase was correlated with the curve of nitrite; it reached its maximum (~60 relative to *rpoD*) when nitrite peaked (bold arrow Fig. 5C) and decreased with its consumption during the stationary phase of growth. Unexpectedly, under the Mn(IV) condition, there are expression levels of these genes that are known to be characteristic of nitrate and nitrite respirations during the exponential phase of growth. At the end of the exponential phase of growth (Fig. 5B,D), the expression level of *napA* reached the same level as that found under the nitrate conditions (~250 relative to *rpoD*). For the *nrfA* expression level, an induction in the Mn(IV) containing cultures was also observed during the exponential phase of growth, reaching 2-fold that of the control (fumarate, see Fig. 5G), but remaining 7-fold lower than in the presence of nitrate (Fig. 5D). Induction of *napA* and *nrfA* genes in the presence of Mn(IV), and the difference in the expression level between fumarate and Mn(IV), are in agreement with a production of nitrate/nitrite that would induce the expression of *napA* and *nrfA*.

The expression level of the *nrfA-2* gene was also investigated. Under the nitrate condition, its overall level was lower than that of *nrfA* (maximal expression level for *nrfA-2* and *nrfA* relative *to rpoD* reached ~6 versus ~60, respectively) but followed the same trend with a sharp increase in the exponential phase of growth and a sharp decrease in the stationary phase of growth (Fig. 5E). In the control (fumarate, Fig. 5G), the expression of this gene followed the same trend as under the nitrate condition. In contrast, in the presence of Mn(IV), the expression level of *nrfA-2* was higher than that of *nrfA* (expression rate relative to *rpoD* ~15 versus ~8, respectively, for *nrfA-2* and *nrfA* for the maximum expression level) (Fig. 5F). At the beginning and during the stationary phase of growth, the expression of *nrfA-2* was 1.9 and 2.9-fold higher than under the nitrate condition, suggesting the maintenance of its role.

To verify that the Mn(IV) induction of genes involved in the N cycle (*nrfA, napA*) leads to the effective reduction of nitrate and nitrite by the bacteria, crude extracts of cells grown in the presence of Mn(IV) were assayed for nitrate and nitrite reductase activities. When fumarate was the terminal electron acceptor in the medium, no significant nitrate or nitrite reductase activity was detected (Table 1). As expected, when cells were grown in the presence of nitrate, both enzymatic activities were measured at an optimal level in the extract and an increase of 443 and 26 fold was observed for the nitrate and the nitrite reductase activities, respectively. Interestingly, the presence of Mn(IV) during the cell growth leads also to a higher level of nitrate and nitrite reductase activity of 150 and 5 fold for the nitrate and nitrite reductase respectively, to those measured in the crude extract of cells grown with fumarate. This result indicates that Mn(IV) induces the production of enzymes able to catalyze nitrate and nitrite reduction. Since our transcriptomic analyses show unambiguously an increase of expression-level of genes involved in nitrate and nitrite respirations, we propose that the measured activities depend on the products of these genes.

Taken together with the induction of *napA* and *nrfA* by nitrate/nitrite observed earlier in *S. oneidensis* MR-1^38^, these data are in agreement with the hypothesis of an intracellular production of nitrite, and possibly nitrate, in *S. algae* C6G3 in the presence of Mn(IV). In the case where nitrate would be produced under the presence of the Mn(IV) condition, the strong expression level of *napA* and the activity of the encoding enzymes could result in the rapid reduction of nitrate, preventing its detection. In contrast, the lower and delayed expression level of *nrfA* in the presence of Mn(IV) could favor the nitrite accumulation observed in physiologic studies. The localization of *nrfA-2* close to NAP-α on the *S. algae* C6G3 genome, together with its higher expression level under the Mn(IV) condition, are in agreement with a direct or indirect role in the production of nitrite under the Mn(IV) condition. Both physiologic transcriptomic and enzymatic data suggest nitrite production at the expense of ammonium in anaerobiosis and in the presence of Mn(IV). Many Bacteria, Archaea and Fungi are known to oxidize ammonium into nitrite by a pathway called nitrification, oxygen being the electron donor for the prokaryote^41^. Bacterial and archaeal autotrophic nitrification requires the concerted action of an ammonia monooxygenase (*amo*) and hydroxylamine oxidase (*hao*). Fungal nitrification is not an energetic pathway and involves the reaction of reduced organic compounds with hydroxyl radicals produced in the presence of hydrogen peroxide and superoxide. Our data show anaerobic ammonium oxidation in the presence of manganese oxide, whereas neither *amo* nor *hao* genes are found on the genome of *S. algae*, suggesting other mechanisms. Several hypotheses could be issued: (i) the presence of an unidentified new gene able to perform ammonium oxidation; (ii) the reverse reaction by an enzyme, such as NrfA or NrfA-2 for example, due to the electron flow equilibrium; and (iii) a secondary reaction generated by a putative oxidative stress.

### Expression of OMC genes in the presence of nitrate or Mn(IV) as the electron acceptor

**Figure 6:**
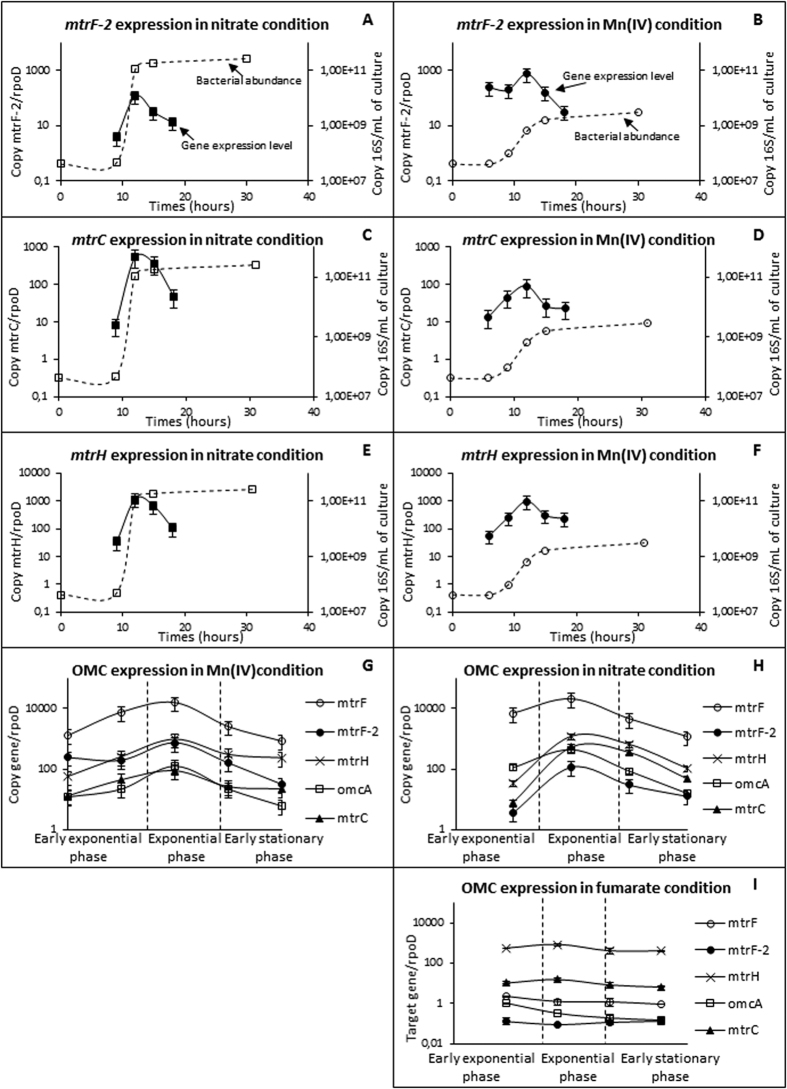
(**A–F)** Outer membrane Cytochrome *(OMC)* gene expression levels*. mtrF-2, mtrC and mtrH* gene expression level along bacterial growth with nitrate or manganese as the electron acceptor (**A–F**). Comparison of expression of 5 OMC in nitrate, Mn(IV) and fumarate condition (negative control) (**G–I**). Standard deviation of growth curves has not been materialized for readability.

To better understand the relative importance of the outer membrane cytochrome in Mn(IV) respiration, comparative monitoring of their gene expression levels was performed between the nitrate and Mn(IV) cultures for the entire growth period (Fig. 6) with fumarate as a control. Mn(IV) reduction is performed in two steps (i) reduction of Mn(IV) to Mn(III) and (ii) reduction of Mn(III) to Mn^2+^, both steps seem to be dependent on the Mtr system, but only the second step causes CO_2_-production^42^.

In the control, the expression levels of all OMC genes were relatively stable during growth, with *mtrF-2* below 0.1, *omcA* and *mtrF* approximately 1, *mtrC* approximately 10 and *mtrH* approximately 600 (relative to *rpoD*, see Fig. 6G–I). In the presence of nitrate or Mn(IV), their expression level increased during the exponential phase of growth then decreased during the stationary phase of growth, except for *mtrH* and *mtrC,* which remained relatively stable at the end of the growth on Mn(IV). The highest expressed gene is *mtrF* (expression rate between 10^3^ to 10^4^) and its expression profiles for nitrate and Mn(IV) conditions are strictly superposed (Fig. 6G,H). Among the cytochrome genes under both conditions, *mtrH* has the second highest expression level after *mtrF* (Fig. 6). The *omcA* gene appeared highly expressed in nitrate compared with Mn(IV) during the entire growth period. In contrast, the *mtrC* and *mtrH* gene expression levels were higher in the Mn(IV) condition only in the early exponential phase (Fig. 6). The *S. oneidensis* MR-1 mutant analysis has shown that *omcA* is not essential for Mn(IV) respiration, whereas *mtrC* could be specific for the second step of Mn(IV) respiration (e.g., the reduction of Mn(III) in Mn^2+^)^43^. Furthermore, each gene has separate promoters, with *omcA* transcribed alone whereas *mtrC* is included in the *mtrCAB* operon^44^. Our results for *S. algae* C6G3 are consistent with these finding. The non-specificity of *omcA* is strengthened by having the same range of expression levels in the nitrate and Mn(IV) cultures with a slightly higher value for the nitrate (Fig. 6G,H). The putative specificity of *mtrC* in the Mn(IV) respiration suggested before is strengthened by our results showing that *mtrC* is significantly over-expressed in the early exponential phase (where Mn^2+^ concentration reached 500 μM) in the manganese oxide relative to the nitrate condition (Fig. 6C,D).

The gene *mtrF-2* (homolog of SO1659 in *S. oneidensis* MR-1 genome) is significantly over-expressed in the presence of Mn(IV) versus nitrate all during growth. At its maximal expression level, it is 6-fold higher for the Mn(IV) condition (Fig. 6A,B) compared with nitrate and 5000-fold higher compared with fumarate (Fig. 6I). Furthermore, low differences between the early-exponential phase and the beginning of the stationary phase of growth of *S. algae* C6G3 in the Mn(IV) condition were observed. From a relative point of view, the OMC expression level profiles show a higher expression level of *mtrF-2* vs. *mtrC* and *omcA* in the Mn(IV) condition whereas in the nitrate condition, *mtrF-2* is less expressed than *mtrC* and *omcA* (Fig. 6G,H). The role of the OMC *mtrF-2* (not belonging to the Mtr operon), is still unclear. Bucking *et al.,* (2010) showed that SO_1659_strep_, a ΔOMC mutant complemented with SO1659 (*mtrF-2*), was unable to restore the rate of Fe(III) and Mn(IV) reduction^45^. Previous studies have shown no significant impact on the respiration of Mn(IV) in *S. oneidensis* MR-1 wild type versus ΔSO1659 (Δ*mtrF-2*)^46^,^47^ at the beginning and at the end of growth. However, the absence of an impact could be due to the colorimetric technique used for the Mn(IV) measures. Because the color of the compound generated is indifferent to the forms of Mn(III) to Mn(VII)^48^, the first step of the reduction of Mn(IV) (Mn(IV) to Mn(III)) could not be monitored. The over-expression level of *mtrF-2* in the presence of Mn(IV), together with the low difference of its expression level between the early-exponential and exponential stages of growth suggest a role in the reduction of Mn(IV) and putatively in the Mn(IV) to Mn(III) reduction step.

The regulation of the OMC expression genes belonging to the Mtr operon seems complex and presents differences between them despite their good synteny, except for *mtrF-2*. Growth phase appeared as an important parameter for the analysis of expression levels of these genes.

## Conclusion

Due to mineralization of organic matter, ammonium concentration present usually higher concentration than that of nitrate or nitrite in sediment, with and increasing tendency with deep, whereas nitrate. nitrite or Mn(IV) show peaks due to net balance between oxidative and reductive process^1^. Physiologic and transcriptomic analysis of *S. algae* C6G3 originating from marine sediment revealed new features regarding nitrate respiration and Mn(IV) and connection between manganese and nitrogen cycles.

Regarding the effectors of the manganese oxide respiration, the data from this study raised interesting points. The *omcA* and *mtrF* genes do not seem specifically involved in Mn(IV) respiration. In contrast, the *mtrC* gene showed a specific induction compared to the nitrate condition, and their products may be involved in the second stage of manganese oxide respiration, which is Mn(III) → Mn^2+^. The clearest and most surprising result of this study is the overexpression under the Mn(IV) condition, throughout the entire growth period, of the *mtrF-2* gene, which does not belong to the Mtr operon. Its strong induction at the beginning and middle of the exponential phase supports the hypothesis of a specific role in the first step of the reduction of manganese oxide which is Mn(IV) → Mn(III).

Regarding connection between nitrogen and manganese cycles, results of this study showed an anaerobic nitrite production in the presence of Mn(IV), with ammonium as the sole nitrogen source. This phenomenon has been already observed *in situ* and in a laboratory microcosm, but this is the first time that this original process has been monitored with a single microorganism. The low nitrite concentration and the absence of nitrate detection is explained by the capacity of dissimilatory reduction of nitrate into ammonium of *S. algae* C6G3. The high-level expression of the *napA* gene in the nitrate condition (where nitrate is reduced into nitrite) and in Mn(IV) condition at the end of the exponential phase of growth indicates the quick reduction of nitrate into nitrite, that is also confirmed by enzymatic nitrate reduction data. Although the expression level of the *nrfA* gene in the Mn(IV) condition does not reach that detected under nitrate condition, the expression level of this gene throughout the experiment and the enzymatic data of nitrite reduction in the Mn(IV) condition could explain the low nitrite concentrations detected. All of these results provide evidence for NO_2/3_^^−^^ production. The role of the *nrfA-2* gene, or of another gene, is one hypothesis for this anaerobic production of nitrite in the presence of Mn(IV), together with the putative reverse reaction of an enzyme such as NrfA or NrfA-2, for example due to electron flow equilibrium or a possible secondary reaction generated by a putative oxidative stress.

## Materials and Methods

### Growth conditions

*S. oneidensis* MR-1 was grown anaerobically at 28 °C in a medium containing 86 mM of NaCl, 20 mM of NH_4_Cl, 50 mM of MgSO_4_, 0.43 mM of K_2_HPO_4_, 13.2 μM of FeSO_4_ and a trace mineral and vitamin solution^49^ and with Mn(IV) (1 mM) as the sole source of electron acceptors and with DL-lactate (25 mM) as a carbon and energy source.

*S. algae* C6G3 and *S. algae* ATCC 51192 were grown anaerobically at 28 °C on artificial sea water^50^ with DL-lactate (25 mM), K_2_HPO_4_ (0.43 mM), FeSO_4_ (13,2 μM) and trace mineral and vitamin solution^49^. This medium contains NH_4_^+^ at 20 mM. Yeast extract (0.01%) was added only in the cultures dedicated for transcriptomic analysis because it allows increasing growth yield without affecting the metabolism (data not shown). The chosen Mn(IV) concentration is a compromise between growth yield and RNA quality because Mn(IV) favor growth, but Mn^2+^ affects RNA integrity. Nitrate (2.5 mM), Mn(IV) (from 0.05 mM to 6 mM), iron oxide (1 or 3 mM) or fumarate (2.5 mM), in the control, was added as the sole source of electron acceptors. No detectable trace of nitrite or nitrate was observed in the Mn(IV), iron oxide or fumarate initial mediums. Manganese oxide was synthesized just before the beginning of the culture following the protocol of Laha *et al.,* 1990^51^ to insure its bioavailability. Iron oxide was also synthesized just before growth, following the protocol of Misawa *et al.,* (1973)^52^. The media were inoculated with the aerobic pre-culture at a ratio of 1:50 in the early exponential phase (OD_600nm_ ~0.020). Anaerobiosis was obtained with a 10-min gas flush (N_2_). All cultures were prepared at least in duplicate.

### Kinetics of growth

Due to the opacity of the culture in the presence of the metal oxides, the abundance of *Shewanella* in the different cultures was determined by q-PCR. The increase of cellular abundance was monitored through the increase of genome number that has been measured according to the increase of the number of 16 S rDNA genes. On cultures with nitrate as electron acceptor, both OD_600_ and 16 S rDNA genes number were determined during growth. Optical density appeared to be correlated with the log of 16 S rDNA genes, with a R^2^ of 0.95. Real time PCR reactions (20 μL) contained 2 μL of diluted culture (1:10) in molecular biology grade water (5PRIME), 0.5 μM of each 16 S primer (see Table S1 in the supplemental material) and 10 μL of SsoAdvancedTM SYBR^®^ Green Supermix (Biorad Lab, USA) and were performed in duplicate on CFX96 Real Time System (C1000 thermal cycler, Bio-Rad Lab, USA) with the following reaction parameters: 2 min at 98 °C, 30 amplification cycles with 5 sec at 98 °C, 10 sec at 57 °C, 20 sec at 72 °C, followed by the melting curve step (between 65 °C to 95 °C, increase of 0.5 °C with a time step of 5 sec) to check the specificity of the amplification product. The absolute quantifications were performed with a standard curve obtained from the dilutions of a DNA plasmid, harboring a ribosomic gene of known concentration as described previously^53^.

The nitrite and nitrate concentrations during growth were measured on an Auto-Analyzer according to Treguer and Le Corre instruction’s^54^. The threshold concentration for nitrate or nitrite is 0.2 μM. The dissolved manganese (Mn^2+^) assay was carried out by colorimetry following the protocol of Chin *et al.,* (1992) with a threshold concentration of 1 μM^55^.

### RNA extraction

The samplings for RNA extractions were carried out at three points of the time series: the early exponential phase, the exponential phase and at the beginning of the stationary phase of growth. Fifteen milliliters of culture were centrifuged for 15 min at 4500 rpm. Cell pellets were suspended in 350 μL of RLT buffer of RNeasy^®^ kit (Qiagen) containing 3.5 μL (1%) of 2-Mercaptoethanol (Aldrich) and were frozen in liquid nitrogen and then conserved at −80 °C until extraction. Then, the total RNA was purified using RNeasy^®^ Mini QIAcube Kit (Qiagen) using the protocol “Purification of total RNA from animal tissues and cells” (eluted in 30 μL, molecular biology grade water) and the DNA contamination was removed using the TURBO DNA-free^TM^ kit (Ambion^®^ by Life Technologies^TM^) according to the manufacturer’s instructions. The RNA quantification and ratio ^260^/_230_ and ^260^/_280_ were measured by spectrophotometry (NanoDrop 2000c Thermo Scientific). The absence of DNA contamination was verified on RNA samples by PCR in RT conditions (1 ng of total RNA per reaction) on the *mtrF* gene (as described below).

### Q(RT)-PCR

Reverse transcription reactions were carried out on 1 ng of total RNA using SuperScript^TM^ III Reverse Transcriptase (Life technologies) with a random primer (250 ng per reaction) according to the manufacturer’s instructions. Dilutions of 1:10 of synthesized cDNAs were used for Q-PCR. The q-PCR primers are listed in Table S1 in the supplemental material. The primers were chosen according to the bibliography and modified according to the sequence of our strain or were newly designed with the help of a primer-designing tool (Primer3 and BLAST, NCBI). For standard curve elaboration, the fragments of genes were amplified. The PCR reactions (20 μL) contained 5 ng DNA of *S. algae* C6G3, 1.25 μM of each primer and 10 μL of Taq’Ozyme Purple Mix 2 (Ozyme) with the following reaction parameters: 2 min at 98 °C, 30 amplifications cycles with 30 sec at 98 °C, 30 sec at 50 °C or 60 °C, 30 sec at 72 °C and ended with 5 min at 72 °C. Each q(RT)-PCR reaction (20 μL) contained 2 μL of cDNAs, 0.5 μM of each primer and 10 μL of SsoAdvanced^TM^ SYBR^®^ Green Supermix (Biorad) and was performed in duplicate on CFX96 Real Time System with the following reaction parameters: 30 sec at 98 °C, 35 two-step amplifications cycles with 5 sec at 98 °C and 10 sec at 62 °C or 58 °C, followed by a melting curve step to verify the specificity of the amplification product. DNA fragments were cloned into pGEMT vector (Promega) and checked by sequencing. The genes detected with RAST^56^ and JGI IMG/ER^57^,^58^ platforms are identified with the accession number of the JGI IMG/ER platform. After purification, the plasmid concentrations were determined by spectrophotometry and used to make the range dilution for the absolute quantification. The starting quantity (SQ) was determined using Bio-Rad CFX Manager 2.1 software and transformed into copies per milliliter of culture according to the sample dilutions. The quantification of the level of expression of the housekeeping gene (*rpoD*) encoding for a σ70 subunit of the RNA polymerase was used to normalize the gene expression. The normalized gene expression was averaged, and the significance of differences in expression was tested with the t-test (XLSTAT version 2010.5.02) and compared.

### Enzymatic activities

Cells of *S. algae* C6G3 were grown anaerobically as described above until the end of the exponential phase of growth. For crude extract preparation, cells were harvested, washed three times in 10 mM TRIS-HCl buffer pH8 containing 86 mM of NaCl and 50 mM of MgSO_4_. Pellets were re-suspended in 50 mM TRIS buffer (pH8) and were disrupted by French press and centrifuged 15 minutes at 13 000 rpm. The crude extract corresponds to the recovered supernatant. The nitrate and nitrite reductase activities present in crude extracts were measured spectrophotometrically at 28 °C by following the oxidation of reduced benzyl viologen at 600 nm coupled to the reduction of nitrate or nitrite, respectively^59^. Activities were expressed as μmol of reduced NO_3_ or NO_2_/min/mg proteins. Protein concentrations were estimated by BioRad Protein assay.

## Additional Information

**How to cite this article**: Aigle, A. *et al*. Physiological and transcriptional approaches reveal connection between nitrogen and manganese cycles in *Shewanella algae* C6G3. *Sci. Rep.* **7**, 44725; doi: 10.1038/srep44725 (2017).

**Publisher's note:** Springer Nature remains neutral with regard to jurisdictional claims in published maps and institutional affiliations.

## Supplementary Material

Supplementary Table S1 and Figures

## Figures and Tables

**Table 1:** Nitrate and nitrite reductase activities of *S. algae* C6G3 crude extract from cells grown in anaerobiosis and with nitrate, Mn(IV) or fumarate as terminal electron acceptors.

Terminal electron acceptor	Nitrate reductase activity (μmol.min^−1^mg prot^−1^)	Nitrite reductase activity (μmol.min^−1^mg prot^−1^)
Nitrate	13.36 ± 3.19	69.58 ± 13.68
Mn(IV)	4.52 ± 0.93	13.34 ± 2.71
Fumarate	0.03 ± 0.03	2.61 ± 0.37
