# Supplementary material for: Physiological and transcriptional approaches reveal connection between nitrogen and manganese cycles in Shewanella algae C6G3
Source: Sci Rep. 2017 Mar 20;7:44725. doi: 10.1038/srep44725 (PMC5357785; doi:10.1038/srep44725)
Supplement: Supplementary Table S1 and Figures [file srep44725-s1.pdf]

Physiological and transcriptional approaches reveal connection between nitrogen and manganese cycles in *Shewanella algae* C6G3

Axel Aigle[1], Patricia Bonin[1], Chantal Iobbi-Nivol[2], Vincent Méjean[2], Valérie Michotey[1]*

Table S1 Presentation of target genes: primers sequences, fragment sizes, annealing temperatures (Tm) and references (ref.).

Primers	Target gene	Primer sequences (5' → 3')	Tm	Fragment size	Ref.
DGGE 300F[1]<br>Univ516R[1]	*16S rDNA*	GCC TAC GGG AGG CAG CAG<br>GTDTTACCGCGGCKGCTGRCA	55 °C	172 pb	(1)
rpoD-F[1]<br>rpoD-R[1]	*rpoD*	TAC GCC GAA GGC CTG AAA AT<br>GAT AGA CAG GCC GGT TTC GG	62 °C	90 pb	This study
nrfA-F1M[2]<br>nrfA-R1M[2]	*nrfA*	GC WTG YTG GAG YTG TAA<br>TAG GGC ATG TGA CAG TC	50 °C	505 pb	(2)
nrfA-2FM[1]<br>nrfA-2RM[1]		CAC GAG AAA GGT TCG CCT A<br>CC CAG GGT CTC CAT GGC	58 °C	68 pb	(3)
nrfA-2-F[2]<br>nrfA-2-R[2]	*nrfA-2*	TGC CAA GGA GTA CCA TAG CC<br>AC AAC TGA TAC CAG CCT TGG	60 °C	547 pb	This study
nrfA-2-q-F[1]<br>nrfA-2-q-R[1]		ACT GAC ACT GCC AAG ACC TC<br>GCA AAC CTG TGA TTG CTG CAT	62 °C	97 pb	This study
napA V66F[2]<br>napA V67R[2]	*napA*	TAYTTYYTNHSNAARATHATGTAYGG<br>DATNGGRTGCATYTCNGCCATRTT	50 °C	414 pb	(4)
napA-3FM[1]<br>napA-3RM[1]		CCC AAC GCC CGT CAC TG<br>CAT GTT GGA GCC CCA AAG	58 °C	130 pb	(3)
mtrC-F[2]<br>mtrC-R[2]	*mtrC*	GGT AAT CCA GGT AAC CCA GG<br>TCT TCT GTC CAC TTG CTG TT	60 °C	856 pb	This study
mtrC-q-F[1]<br>mtrC-q-R[1]		GCT ACA CCT TCA GCA AGA CCT<br>GTA TTG TAA GCG CGC AGC AG	62 °C	97 pb	This study
mtrF-F[2]<br>mtrF-R[2]	*mtrF*	CAT TGC CGG TAA CCA ATG AC<br>TAC TCA GCT TGA GCG AGA AG	60 °C	540 pb	This study
mtrF-q-F[1]<br>mtrF-q-R[1]		CCG GAT TCC CCA AAC CCT TG<br>GCC ACA GGA CTC CAT GGT TG	62 °C	107 pb	This study
mtrF-2-F[2]<br>mtrF-2-R[2]	*mtrF-2*	CTC CTG GAA CGC CAA TAT CT<br>AAC TTA ACG CTT GAA GTG GC	60 °C	581 pb	This study
mtrF-2-q-F[1]<br>mtrF-2-q-R[1]		CCA ATA TCT ACC GCG AAG CCT<br>CTG ACA TCG ACT GTC GTC GG	62 °C	110 pb	This study
omcA-F[2]<br>omcA- R[2]	*omcA*	GGT GCT GGA TAA AGA CGG TA<br>GCA GTG TCT TAT CCG GTG TA	60 °C	506 pb	This study
omcA -q-F[1]<br>omcA -q-R[1]		AGC CGT ATG ATA GTG GGC TG<br>TCA CTG AGA CGA ATA CGG CG	62 °C	89 pb	This study
mtrH-F[2]<br>mtrH-R[2]	*mtrH*	CTA CAG CAA CAA CGA CAG TG<br>TCA TTT CGG CAT GTT GAA CC	60 °C	515 pb	This study
mtrH-q-F[1]<br>mtrH-q-R[1]		TCT GCC AAG ACC AGC GTT AC<br>CCG TGC TTG ACA GTC ATG TTG	62 °C	86 pb	This study

[1]Primers used for Q-PCR analysis, [2]primers used to make clones.

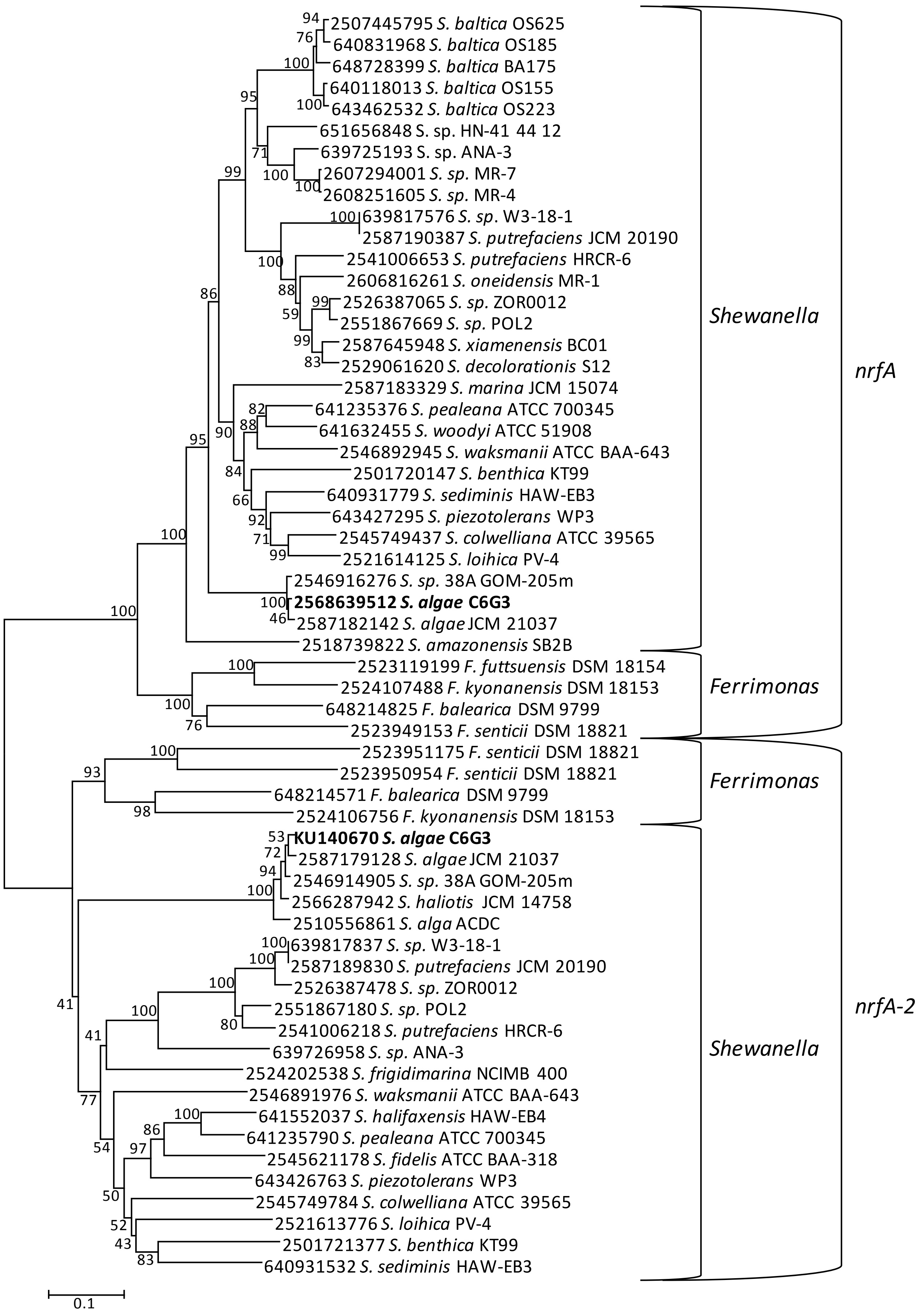

Figure S1 Phylogenetic distribution of NrfA and NrfA-2 through *Shewanella* and *Ferrimonas* genus. This Neighbor-joining tree is based on 747 amino acid characters aligned. The bootstrap percentages higher than 50% are indicated at the node after 1000 resampled data sets. The branch length corresponds to the sequence differences as indicated on the scale bar (substitutions per position).

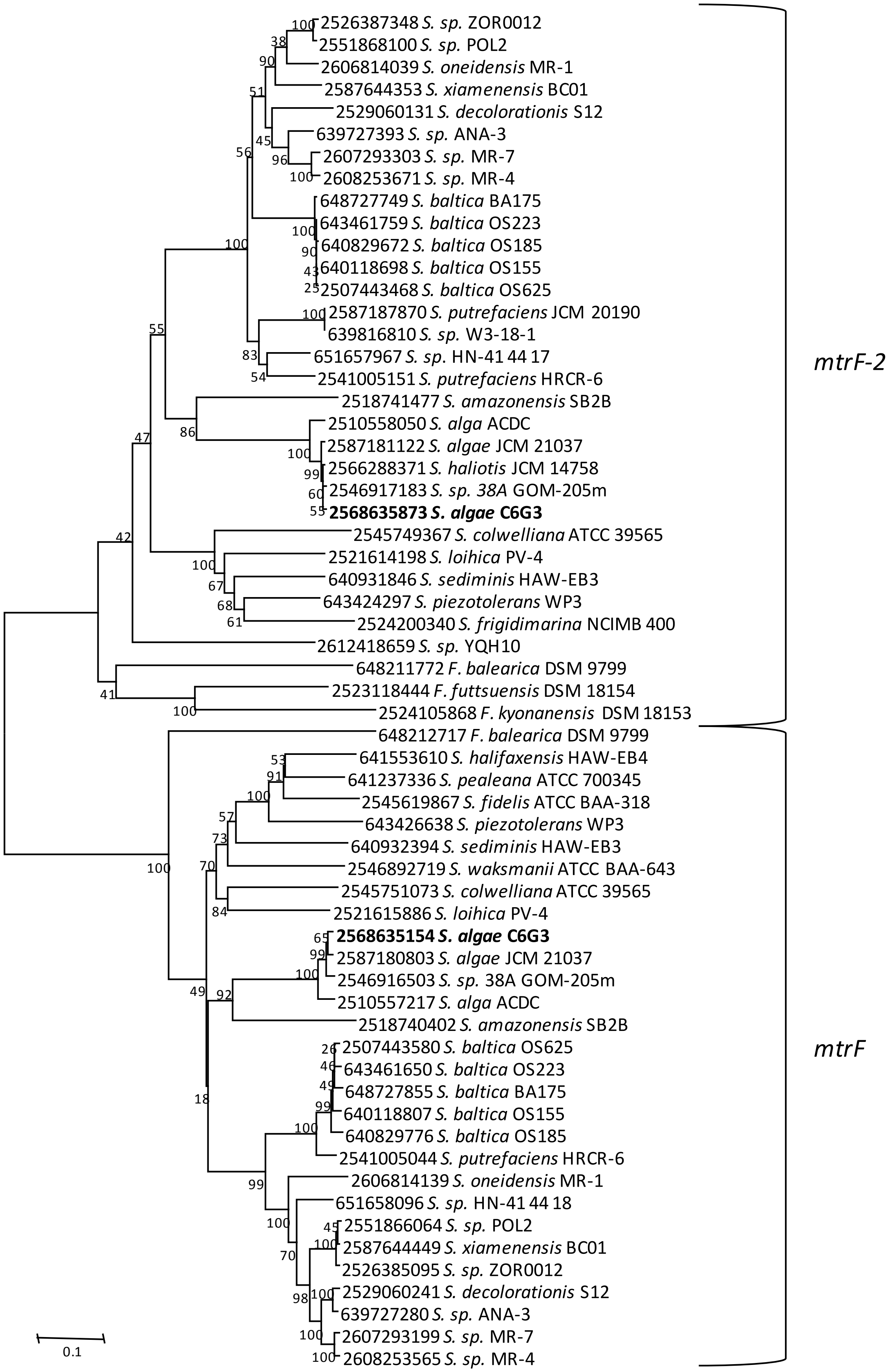

Figure S2 Phylogenetic distribution of MtrF and MtrF-2 through *Shewanella* and *Ferrimonas* genus. This Neighbor-joining tree is based on 2,178 amino acid characters aligned. The bootstrap percentages higher than 50% are indicated at the node after 1000 resampled data sets. The branch length corresponds to the sequence differences as indicated on the scale bar (substitutions per position).

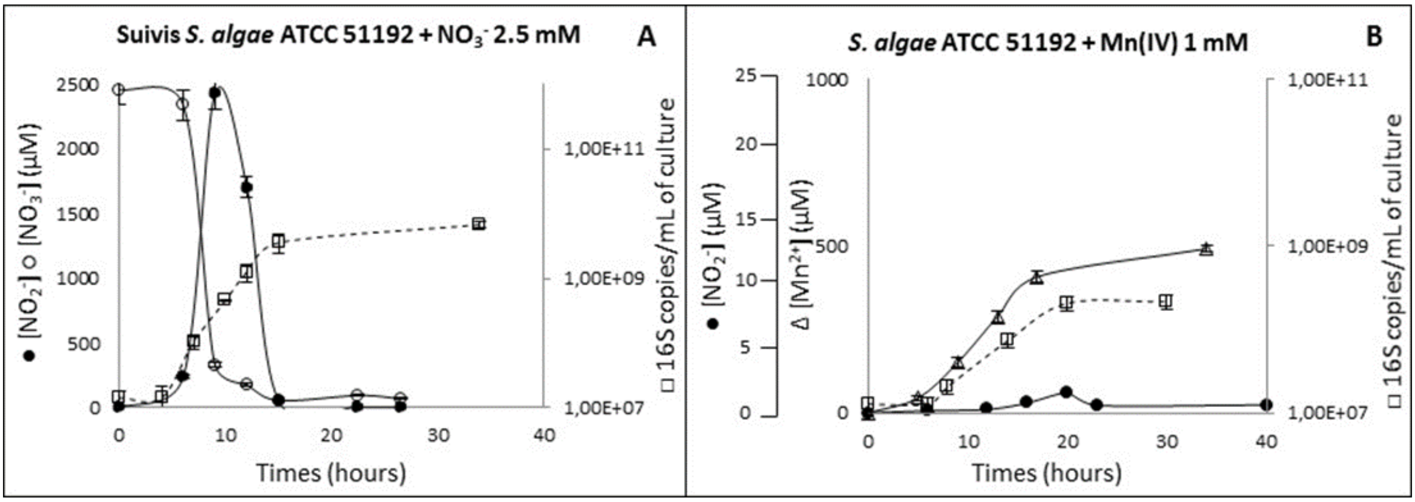

Figure S3 Follow-ups of *S. algae* ATCC 51192 growth (□) on nitrate (3A) or manganese oxide (3B) and concentrations of dissolved nitrite (●), nitrate (○) and manganese (Δ).

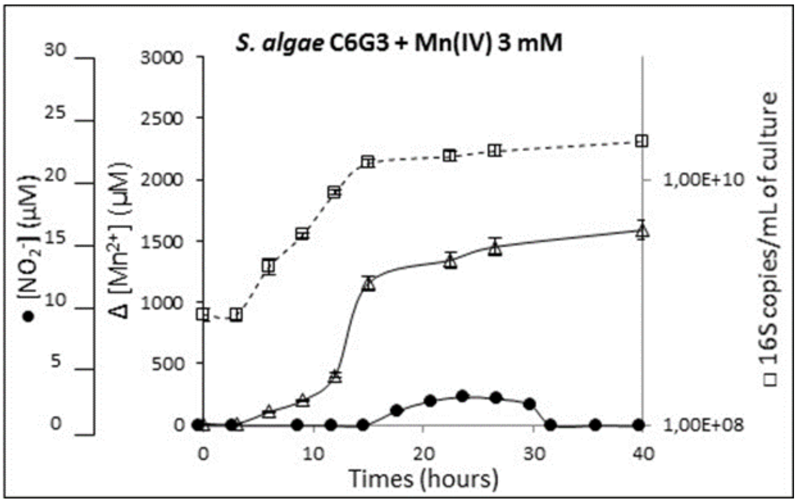

Figure S4. Follow-ups of the bacterial growth (□) and concentrations of dissolved nitrite (●) and manganese (Δ).

References

1. **Casamayor EO, Massana R, Benlloch S, Øvreås L, Díez B, Goddard VJ, Gasol JM, Joint I, Rodríguez-Valera F, Pedrós-Alió C.** 2002. Changes in archaeal, bacterial and eukaryal assemblages along a

salinity gradient by comparison of genetic fingerprinting methods in a multipond solar saltern. Environmental Microbiology **4:**338-348.

2. **Mohan SB, Schmid M, Jetten M, Cole J.** 2004. Detection and widespread distribution of the nrfA gene encoding nitrite reduction to ammonia, a short circuit in the biological nitrogen cycle that competes with denitrification. Fems Microbiology Ecology **49:**433-443.
3. **Smith CJ, Nedwell DB, Dong LF, Osborn AM.** 2007. Diversity and abundance of nitrate reductase genes (narG and napA), nitrite reductase genes (nirS and nrfA), and their transcripts in estuarine sediments. Applied and Environmental Microbiology **73:**3612-3622.
4. **Nogales B, Timmis KN, Nedwell DB, Osborn AM.** 2002. Detection and diversity of expressed denitrification genes in estuarine sediments after reverse transcription-PCR amplification from mRNA. Applied and Environmental Microbiology **68:**5017-5025.
